# Avoiding Unnecessary Urethroplasty by Periurethral Mobilization as a Treatment Option for Small Urethral Strictures: A Case Series

**DOI:** 10.7759/cureus.95414

**Published:** 2025-10-25

**Authors:** Lalit Kumar, Singathala Gnana Sree, Yashasvi Singh, Ujwal Kumar, Sameer Trivedi

**Affiliations:** 1 Department of Urology, Institute of Medical Sciences, Banaras Hindu University, Varanasi, IND

**Keywords:** adhesiolysis, intraoperative reassessment, periurethral mobilization, urethral stricture, urethroplasty

## Abstract

Urethral stricture remains a frequently encountered urological condition, with urethroplasty often regarded as the standard approach for complex or recurrent cases. However, in specific scenarios, intraoperative assessment may reveal less severe pathology, allowing for a more conservative and less invasive management strategy. This case series describes male patients who were scheduled for urethroplasty but were ultimately treated successfully with periurethral mobilization, adhesiolysis, and catheterization alone. Four male patients, aged between 19 and 51 years (mean age 31 years), presented with lower urinary tract symptoms, including weak urinary stream, straining, incomplete emptying, and acute urinary retention. Preoperative imaging with retrograde urethrogram and micturating cystourethrogram suggested bulbar urethral strictures with stricture length ranging from 0.5 to 2cm (mean 1.5 cm), and all patients were planned for urethroplasty. Initial maximum urinary flow rates (Qmax) ranged from 2 to 6 mL/second, with a mean of 3.75 mL/second, while post-void residual (PVR) urine volumes ranged from 200 to 320 mL, with a mean of 237.5 mL.

Intraoperatively, after mobilizing the urethra, dense periurethral adhesions were observed without significant intraluminal narrowing. Gentle insertion of a 16 Fr Foley catheter was successful in each case, leading to the decision to avoid urethroplasty. All patients were managed with adhesiolysis and catheter drainage. Postoperative follow-up demonstrated marked improvement in symptoms, with Qmax ranging from 24 to 27 mL/second, with a mean of 25.25 mL/second, and PVR volumes decreasing to 10 to 15 mL, with a mean of 12.75 mL. This series highlights the importance of intraoperative re-evaluation in patients with suspected urethral stricture. Careful mobilization and catheter testing can help identify those who can be effectively managed without urethroplasty, thereby minimizing surgical morbidity. Incorporating and reinforcing this step into operative planning can support more individualized and less invasive treatment strategies.

## Introduction

Urethral stricture disease is a fixed anatomical narrowing of the urethral lumen caused by fibrosis of the corpus spongiosum, resulting in impaired urinary flow and lower urinary tract symptoms [1]. It most commonly affects adult males and predominantly involves the bulbar urethra [2]. The reported prevalence ranges from 0.2% to 0.6% (229-627 cases per 100,000 males), with incidence increasing with age [3]. The condition may result from trauma, iatrogenic causes such as repeated catheterization or endoscopic procedures, infections (including sexually transmitted diseases), inflammatory disorders such as lichen sclerosus, or may be idiopathic [4,5]. Common presenting symptoms include weak urinary stream, straining, post-void dribbling, increased frequency, and incomplete bladder emptying. If untreated, complications such as recurrent urinary tract infections, bladder stones, diverticula, and renal impairment can occur [4]. Diagnostic evaluation typically includes uroflowmetry, post-void residual (PVR) urine measurement, retrograde urethrogram (RGU), and micturating cystourethrogram (MCU) [6,7].

Recent studies indicate that approximately 6-10% of patients initially planned for urethroplasty are found intra-operatively to have short, passable strictures that can be managed conservatively without reconstruction [8]. Recognizing such cases can help avoid unnecessary urethroplasty and its associated morbidity [9]. According to the latest European Association of Urology (EAU) 2024 and American Urological Association (AUA) 2023 guidelines, mild cases can be managed by endoscopic visual internal urethrotomy (VIU) or dilatation, which achieves up to 80% success in first-time short (<1 cm) bulbar strictures [1,10]. However, urethroplasty remains the gold standard for longer, recurrent, or complex strictures. Excision-and-primary-anastomosis urethroplasty is preferred for short (<2 cm) bulbar strictures, while substitution or augmentation urethroplasty using buccal mucosa or penile/preputial flaps is reserved for longer or complex lesions [11].

This case series highlights the importance of intraoperative re-evaluation to identify patients who may be successfully treated by peri-urethral mobilization and adhesiolysis, thereby avoiding unnecessary urethroplasty. Our experience aligns with current EAU and AUA guidelines emphasizing individualized, anatomy-based surgical decision-making [1,10].

## Case presentation

This case series includes four male patients who presented to the Urology Outpatient Department over the last 1.5 years with symptoms of urethral stricture. Ages ranged from 19 to 51 years (mean 31 years). All patients reported varying durations of lower urinary tract symptoms such as poor urinary stream, straining, intermittent flow, and incomplete voiding. RGU and MCU revealed bulbar urethral strictures. Preoperative uroflowmetry showed maximum urinary flow rates (Qmax) ranging from 2 to 6 mL/second (mean 3.75 mL/second) compared to normal values >15 mL/second, and PVR urine volume ranged from 200 to 320 mL (mean 237.5 mL) compared to a normal range of <50 mL.

**Figure 1:**
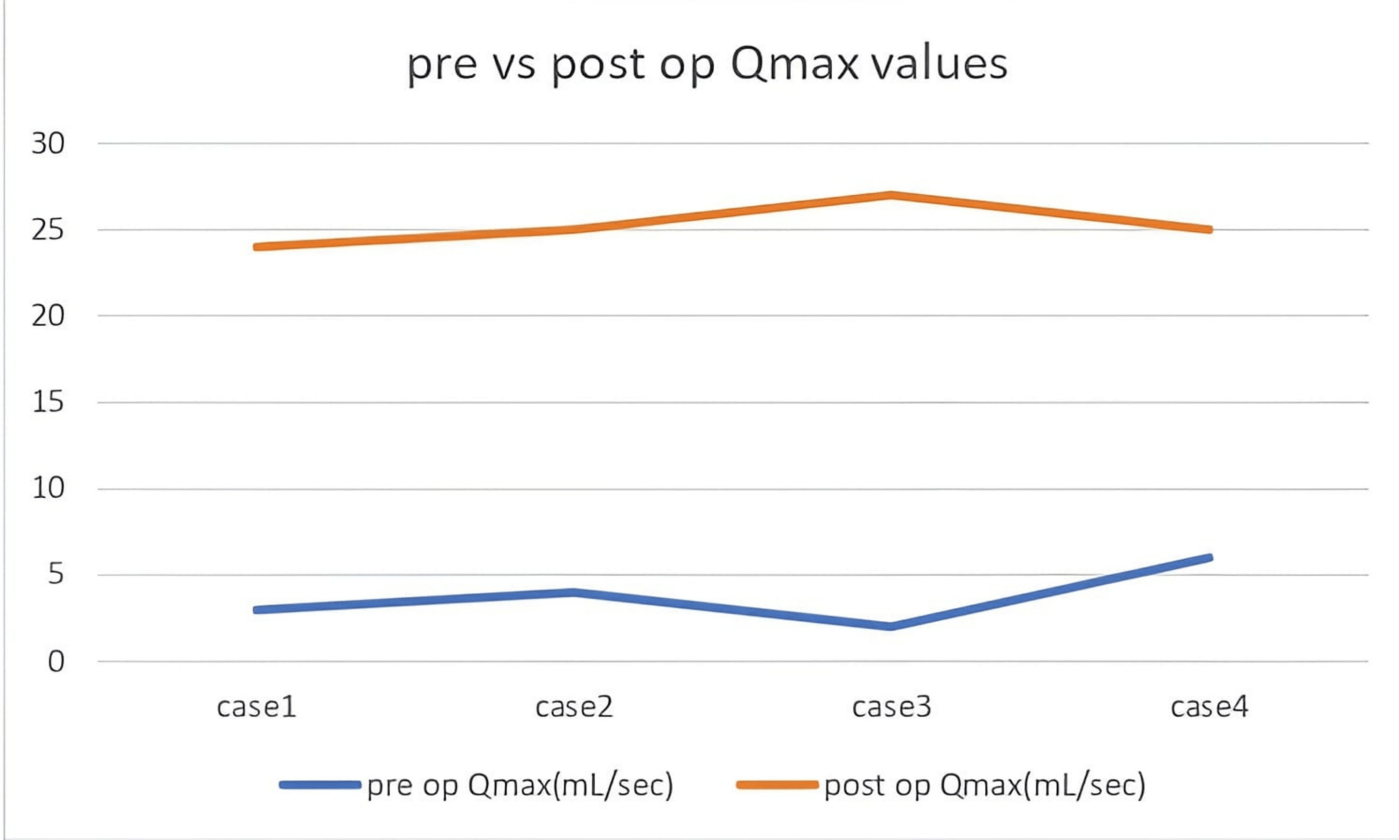
Preoperative vs. postoperative Qmax values.

**Figure 2:**
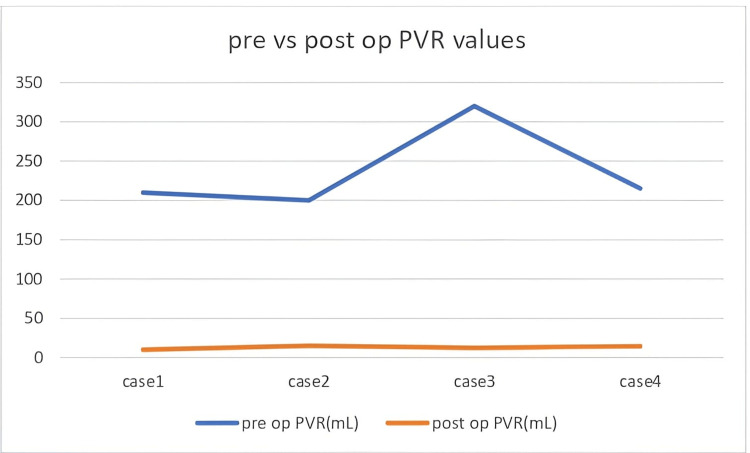
Preoperative vs. postoperative post-void residual (PVR) values.

Initial treatment plans involved urethroplasty in all cases after discussing surgical options with patients. However, intraoperative assessment following periurethral mobilization revealed that all strictures were passable upon gentle catheterization with a 16 Fr Foley catheter. As a result, patients were managed with periurethral adhesiolysis and catheter placement only, avoiding formal urethroplasty. There were no intraoperative or postoperative complications in any case. Patients were discharged within 48 hours post-surgery, and per-urethral catheters were removed within five days. Postoperative Qmax ranged from 24 to 27 mL/second (mean 25.25 mL/second), values within the normal range of >15 mL/second, and PVR volumes decreased to 10-15 mL (mean 12.75 mL), returning to the normal limit of <50 mL (Figures 1-2). Follow-up duration ranged from four to 18 months, with a mean of 9.5 months, and was uneventful in all cases (Table 1).

**Table 1:** Patient demographics, clinical presentation, and management overview.

Parameter	Case 1	Case 2	Case 3	Case 4
Age (years)	28	51	19	27
Etiology	Idiopathic	Traumatic catheterization	Idiopathic	Idiopathic
Symptom onset	4 months	4 months	1 year	1.5 years
Primary/recurrent	Primary	Primary	Primary	Primary
Initial diagnosis	Proximal bulbar stricture	Proximal bulbar stricture	Distal bulbar urethral stricture	Mid-bulbar stricture
Stricture length (cm)	0.5	2	2	1.5
Preoperative Qmax (mL/second) (normal >15)	3	4	2	6
Preoperative PVR (mL) (normal <50)	210	200	320	215
Postoperative Qmax (mL/second) (normal >15)	24	25	27	25
Postoperative PVR (mL) (normal <50)	10	15	12	14
Procedure planned	Non-transecting urethroplasty	Non-transecting urethroplasty	Augmentation urethroplasty	Non-transecting urethroplasty
Procedure performed	Adhesiolysis	Adhesiolysis	Adhesiolysis	Adhesiolysis
Perioperative complications	None	None	None	None
Follow-up	4 months	4 months	1 year	1.5 years

Inclusion criteria for this series included single, short-segment (<2 cm), primary bulbar urethral strictures confirmed on RGU and MCU. Pre-operative cystourethroscopy in all patients revealed complete luminal blockage consistent with radiologic findings. However, intra-operatively, after careful peri-urethral mobilization and adhesiolysis, a 16 Fr Foley catheter could be passed easily into the bladder, confirming that the obstruction was due to external peri-urethral adhesions rather than dense intraluminal spongiofibrosis. Our institution does not routinely perform penile ultrasound or MRI to assess fibrosis depth as part of the standard management of urethral stricture disease; hence, the degree of fibrosis was determined intra-operatively by direct visualization and palpation.

Case 1

**Figure 3:**
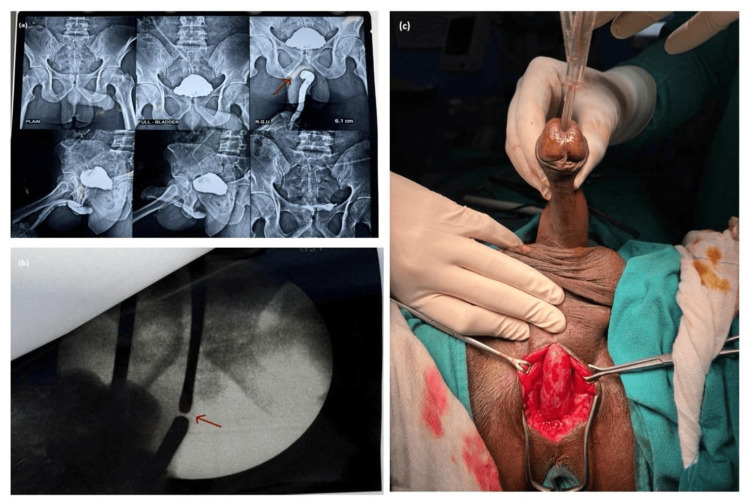
(a) Retrograde urethrogram (RGU)/micturating cystourethrogram (MCU) showing narrowing at proximal bulbar urethra, suggestive of urethral stricture (indicated by red arrow). (b) Bougiegram confirmed the urethral stricture. (c) Intraoperative image showing periurethral mobilization and catheter in urinary bladder.

A 28-year-old male presented to the Urology Outpatient Department with complaints of poor urinary stream, straining, intermittent flow, and a sense of incomplete voiding for the past four months. He developed acute urinary retention for which a suprapubic catheter was placed after failed attempts at urethral catheterization, suggestive of an underlying urethral stricture. RGU and MCU revealed a complete block at the proximal bulbar urethra with a stricture length of 0.5 cm (Figure 3a). Preoperative cystourethroscopy revealed complete luminal blockage corresponding to the radiologic impression. Bougiegram confirmed the findings (Figure 3b). Based on imaging, he was planned for non-transecting bulbar urethroplasty. Preoperative uroflowmetry showed severely reduced urinary flow parameters, with Qmax 3 mL/second and a PVR volume of 210 mL. During surgery, a vertical perineal incision was made, and periurethral dissection was performed. After gentle mobilization, a 16 Fr urethral catheter was successfully passed into the bladder, indicating a patent lumen (Figure 3c). Hence, the decision was made to defer urethroplasty, and only periurethral adhesiolysis was completed. The patient was discharged after 48 hours without any complications with a per-urethral catheter in situ. The catheter was removed within five days. On follow-up at four weeks, uroflowmetry demonstrated significant improvement with a Qmax of 24 mL/second and PVR volume of 10 mL, confirming the functional success of the conservative approach.

Case 2

**Figure 4:**
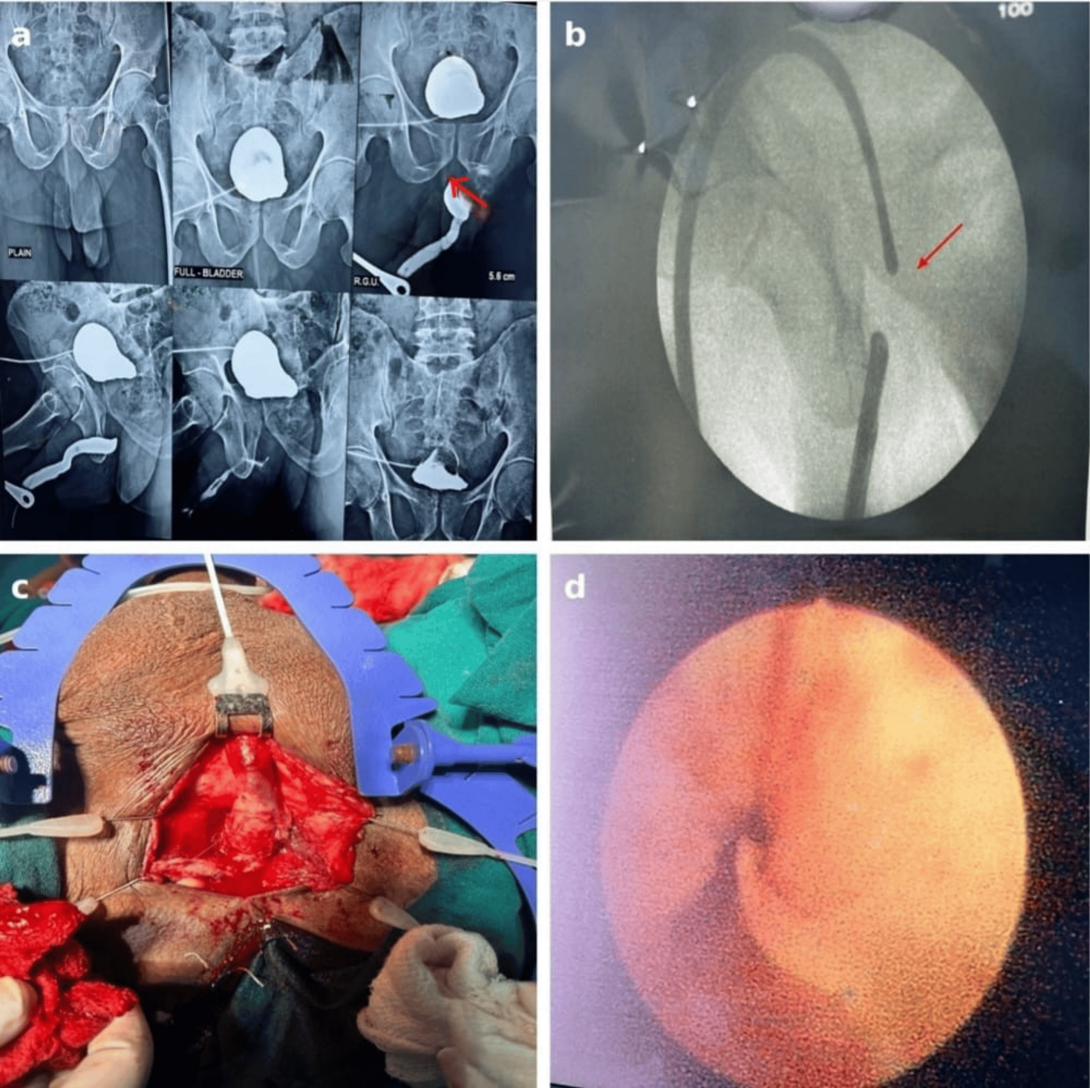
(a) Retrograde urethrogram (RGU)/micturating cystourethrogram (MCU) identifies proximal bulbar urethral stricture (red arrow). (b) Urethral stricture confirmed by bougiegram. (c) Intraoperative image showing bulbar urethral mobilization. (d) On cystourethroscopy, urethral tag was seen, but the cystoscope was easily negotiable into the urinary bladder.

A 51-year-old male presented with complaints of poor urinary stream, straining, intermittent flow, and a sensation of incomplete bladder emptying for the past four months. He had a history of traumatic urethral catheterization, following which he developed acute urinary retention and underwent suprapubic catheter placement at an outside hospital. The inability to negotiate the catheter through the urethra indicated an underlying urethral obstruction. RGU and MCU showed a complete block in the proximal bulbar urethra with a stricture length of approximately 2 cm (Figure 4a). Cystourethroscopy performed preoperatively showed total occlusion of the lumen. Bougiegram confirmed the presence of a stricture (Figure 4b). Preoperative uroflowmetry showed Qmax of 4 mL/second and PVR volume of 200 mL. Based on these findings, he was initially scheduled for non-transecting urethroplasty. Intraoperatively, through a vertical perineal approach, periurethral adhesiolysis was carried out. Upon gentle urethral mobilization, a 16 Fr Foley catheter was successfully passed into the bladder without resistance (Figure 4c). This indicated adequate patency and led to deferral of the planned non-transecting urethroplasty. On cystoscopy, a urethral tag was observed, possibly due to fibrosis (Figure 4d). No additional surgical intervention was deemed necessary. The patient did well and was discharged two days after surgery with a per-urethral catheter in place, which was removed within five days. On follow-up after four weeks, uroflowmetry demonstrated significant clinical improvement with a post-operative Qmax of 25 mL/second and PVR volume of 15 mL, confirming a successful outcome with conservative management.

Case 3

**Figure 5:**
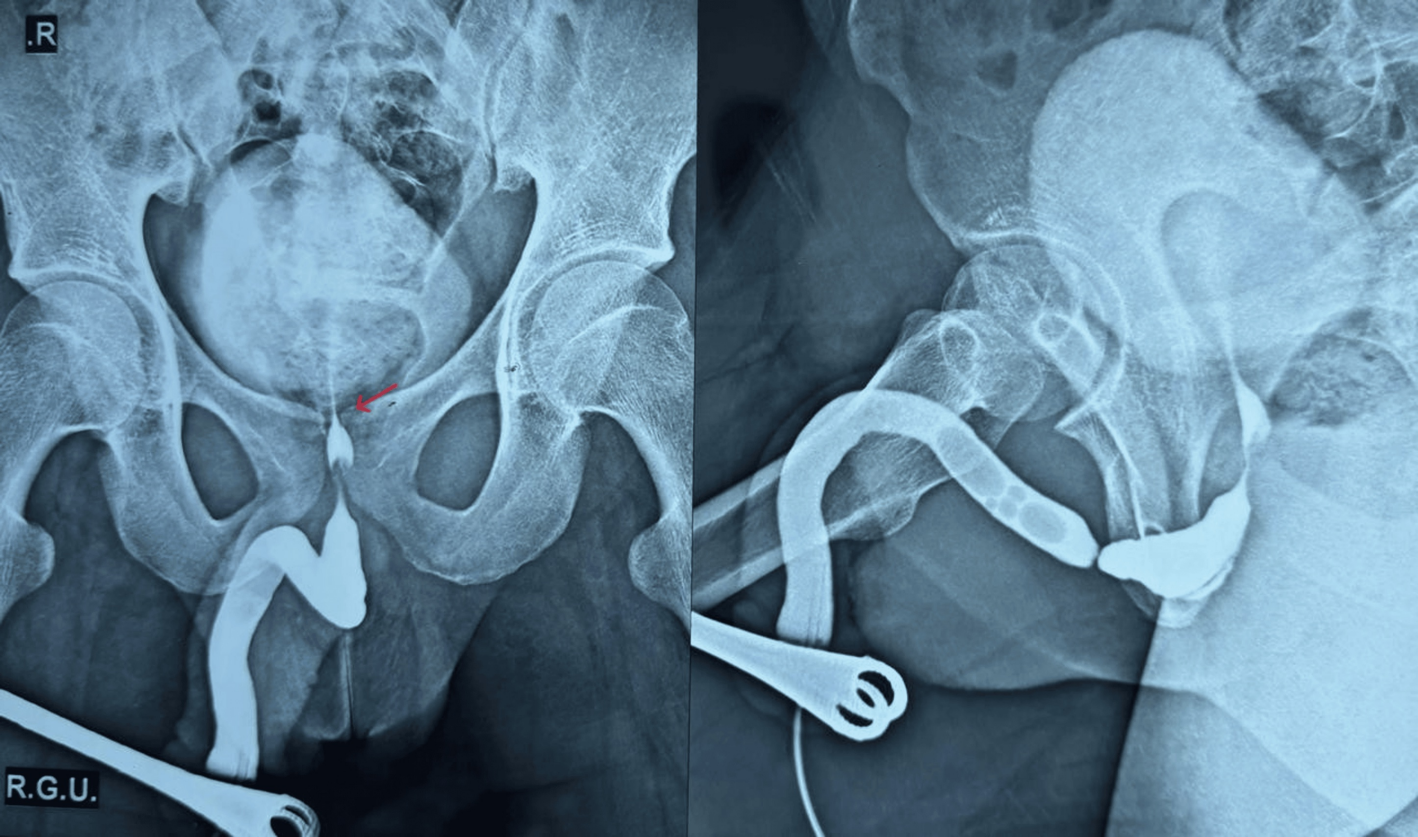
Retrograde urethrogram (RGU) demonstrating distal bulbar urethral stricture shown by red arrow.

A 19-year-old male presented to the Urology Outpatient Department with complaints of poor urinary stream, straining, a sense of incomplete voiding, and intermittency for the past year. There was no history of hematuria, graveluria, flank pain, trauma, or urinary tract infections. The patient had no prior surgeries or comorbidities but reported tobacco use for seven years. Preoperative uroflowmetry revealed a voided volume of 235 mL, a maximum flow rate (Qmax) of 2 mL/second, and a PVR of 220 mL. RGU and MCU showed a tight stricture at the proximal urethra, with an estimated stricture length of 2 cm (Figure 5). On preoperative cystourethroscopy, the lumen appeared completely obstructed. He was initially scheduled for augmentation urethroplasty. However, during intraoperative assessment, following periurethral mobilization and careful adhesiolysis, a 16 Fr per-urethral catheter was able to pass into the bladder without resistance. As the stricture was found to be amenable to simple adhesiolysis, definitive urethroplasty was deferred. The postoperative period was uneventful. At four weeks, uroflowmetry showed a Qmax of 27 mL/second and PVR of 12 mL, indicating satisfactory functional recovery.

Case 4

**Figure 6:**
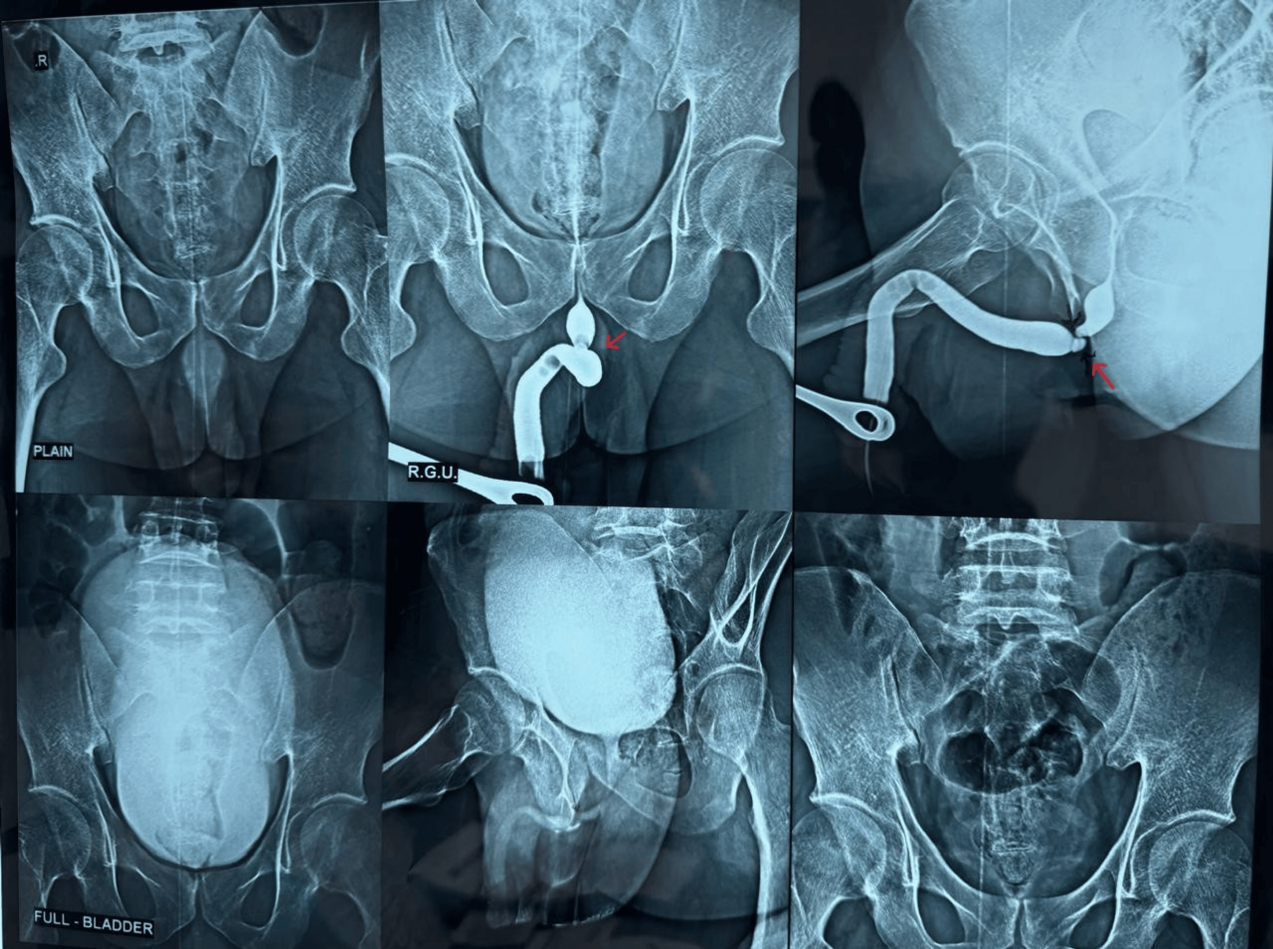
Retrograde urethrogram (RGU) identifying mid-bulbar urethral stricture.

A 27-year-old male presented with complaints of poor urinary stream, straining, incomplete voiding, and intermittency for the past 1.5 years. He denied any history of hematuria, graveluria, flank pain, trauma, or urinary tract infections. There was no prior history of genitourinary surgery or significant comorbidities, although he had a history of tobacco use for seven years. Initial uroflowmetry showed a voided volume of 158 mL, a maximum flow rate (Qmax) of 6 mL/second, and a PVR urine of 320 mL. RGU and MCU revealed a narrowing involving the mid-bulbar urethra with an estimated stricture length of 1.5 cm (Figure 6). Cystourethroscopy before surgery demonstrated complete blockage. The patient was initially scheduled for non-transecting urethroplasty. However, intraoperatively, dense periurethral adhesions were encountered throughout the penobulbar segment, along with areas of spongiofibrosis and unhealthy mucosa extending from the penobulbar junction to the bulbo-membranous urethra. After careful periurethral mobilization and adhesiolysis, a 16 Fr per-urethral catheter was successfully passed into the bladder without resistance, indicating that the stricture was passable and did not require formal reconstructive urethroplasty. The patient tolerated the procedure well and was discharged with a per-urethral catheter in situ. On follow-up, uroflowmetry demonstrated a Qmax of 25 mL/second and a PVR volume of 14 mL, confirming significant functional improvement.

## Discussion

Urethral stricture disease continues to be a significant concern in urology, with the choice of surgical approach being a critical factor in treatment success. Urethroplasty has established itself as the preferred treatment for anterior urethral strictures, demonstrating high long-term efficacy and outperforming other treatments such as dilation and optical internal urethrotomy [1,10]. However, urethroplasty, particularly the transecting and augmentation types, carries inherent risks including wound infection, ischemia, sexual dysfunction, chordee, and perioperative morbidity [4]. Hence, careful intraoperative assessment is essential to avoid overtreatment and to tailor the surgical intervention based on real-time anatomical findings. In this case series, four patients initially scheduled for definitive urethroplasty were instead managed with periurethral adhesiolysis alone, a less invasive technique, guided by intraoperative evaluation. This shift in the treatment strategy was based on findings that the urethral strictures were shorter, passable, and not associated with significant spongiofibrosis or obliteration, contrary to what preoperative imaging suggested.

RGU and MCU are standard diagnostic tools for assessing the length, location, and severity of urethral strictures. However, these modalities can sometimes overestimate or underestimate the actual degree of luminal narrowing, as noted in our patients [6]. RGU, for example, may not differentiate between a dense stricture and a complete obliteration, leading to a more aggressive surgical plan than is warranted. In contrast, intraoperative visualization, along with catheter calibration after mobilizing the urethra, allows for a more reliable assessment of luminal integrity. Careful pre-operative evaluation using RGU and MCU helps delineate the length and site of narrowing, but these modalities may not always distinguish between intraluminal fibrosis and external adhesions. Ultrasound urethrography or MRI can assist in assessing spongiofibrosis depth, but these are not routinely used in our institution. Cystourethroscopy remains the definitive assessment to confirm luminal patency and the nature of the obstruction.

Each patient in our series presented with symptoms consistent with lower urinary tract obstruction, including poor stream, intermittency, and a sensation of incomplete voiding. Preoperative uroflowmetry demonstrated markedly reduced flow rates ranging between 3 and 6 mL/second and elevated PVR urine volumes ranging between 200 and 320 mL, deviating significantly from normal values for Qmax (>15 mL/second) and PVR (<50 mL) [7,12]. Based on radiologic evidence of strictures ranging from 0.5 cm to 2 cm in length located at the bulbar urethra, the surgical plan for each case involved urethroplasty. However, intraoperative mobilization of the urethra allowed passage of a 16 Fr catheter into the bladder without significant resistance in all cases. This clinical maneuver indicated the absence of a complete lumen block, severe spongiofibrosis, or a need for urethral reconstruction, and thus averted potentially unnecessary urethroplasties.

Peri-urethral fibrosis typically results from localized inflammation or prior instrumentation that causes scarring around the urethral wall rather than within the lumen itself. This peri-urethral fibrotic reaction contracts externally, mimicking a true stricture on imaging. During mobilization, gentle adhesiolysis releases these fibrous bands and restores urethral patency while maintaining urethral vascular integrity [8,9]. Periurethral adhesions, particularly in the bulbar and penobulbar regions, can sometimes mimic complete strictures on imaging [4]. Such adhesions can arise from previous inflammation, trauma related to catheterization, or other iatrogenic factors, though in some instances, no clear cause is identified. Intraoperative dissection and mobilization of the periurethral tissues frequently uncover a patent urethral lumen that permits catheter passage, as demonstrated in our patient series. This insight aligns with existing literature emphasizing the role of intraoperative decision-making in tailoring urethral stricture treatment. Furthermore, intraoperative assessment avoids the need for unnecessary urethral transections, which are associated with greater morbidity. Transecting urethroplasty may compromise the neurovascular integrity of the penis and result in erectile dysfunction or loss of penile length [13]. By adopting a conservative yet effective and safe approach in these cases, the surgical team ensured preservation of urethral function and minimized complications.

The postoperative course in all four patients was uneventful. Follow-up uroflowmetry at four weeks post-catheter removal revealed improvement in Qmax from preoperative values of 3 to 6 mL/second to postoperative values of 24 to 27 mL/second, with significant reduction in PVR urine from 200 to 320 mL preoperatively to 10 to 15 mL postoperatively, bringing patient values closer to established normal threshold for Qmax (>15 mL/second) and PVR (<50 mL). These objective findings emphasize the therapeutic adequacy of adhesiolysis in carefully selected cases. It is also important to highlight that preoperative imaging remains vital in surgical planning. However, it must be interpreted with caution, particularly in patients with a history of instrumentation or prolonged indwelling catheters. In these settings, periurethral scarring can lead to exaggerated radiologic impressions of stricture severity [6].

Emerging evidence supports the use of urethral calibration and endoscopic evaluation for dynamic assessment of stricture severity and tissue quality [1,10]. While cystoscopy was not employed in all cases, the intraoperative ability to pass a standard 16 Fr catheter following adhesiolysis served as a reliable surrogate, suggesting that minimal luminal compromise was present. Moreover, the condition of the urethral tissue, specifically the presence of intact mucosa and minimal fibrosis, can influence the decision to adopt a less invasive surgical approach. In the fourth case, despite the presence of unhealthy mucosa, the lumen was navigable after adhesiolysis, and functional outcomes were favorable. This demonstrates that not all mucosal abnormalities necessitate formal urethroplasty, especially if the urethral patency can be maintained. The choice to forego urethroplasty should be guided by a comprehensive intraoperative evaluation that incorporates tactile sensation, direct visual assessment, and catheter-based calibration. This approach aims to ensure the best possible functional outcome while reducing operative morbidity. Our findings add to the growing body of literature that challenges the routine use of reconstructive surgery for all strictures and emphasizes the significance of individualized surgical planning [1,4,10].

In conclusion, this case series demonstrates that intraoperative assessment following urethral mobilization is a critical step in the management of urethral strictures. Adhesiolysis with catheter passage can serve as a definitive treatment in select patients, thereby avoiding the risks associated with urethroplasty. Identifying candidates who may benefit from conservative management rather than urethroplasty remains a challenge. Primary, short-segment (<2 cm) bulbar strictures with smooth tapering on imaging and no dense spongiofibrosis may be ideal for peri-urethral mobilization and adhesiolysis. Being a small case series, inclusion of a control group was not feasible. Hence, larger prospective multicentric studies are required to validate our findings and establish objective pre-operative selection criteria. Larger prospective multicentric studies are needed to validate these findings and potentially redefine surgical guidelines for urethral stricture disease.

## Conclusions

This case series highlights the key role of intraoperative assessment in the management of urethral stricture disease. While all four of our patients were initially scheduled for definitive urethroplasty based on preoperative imaging and clinical presentation, on surgical exploration, the catheter went in easily after periurethral mobilization. Thus, patients with small urethral strictures can be given a trial of gentle urethral catheterization to eliminate the need for reconstructive urethral surgery. These cases emphasize the importance of adopting a dynamic and individualized surgical approach, where decisions are guided not solely by imaging but by real-time intraoperative findings, and adding a step of urethral calibration with a catheter after urethral mobilization. By avoiding unnecessary urethroplasty, the surgical team minimized patient morbidity while achieving good functional outcomes, as demonstrated by improved uroflowmetry and reduced PVR urine on follow-up. Further studies with larger cohorts are needed to validate these observations and potentially refine treatment algorithms for anterior urethral stricture disease.
